# Supplementary material for: Brightness modulations of our nearest terrestrial planet Venus reveal atmospheric super-rotation rather than surface features
Source: Nat Commun. 2020 Nov 12;11:5720. doi: 10.1038/s41467-020-19385-6 (PMC7665209; doi:10.1038/s41467-020-19385-6)
Supplement: Supplementary file 1 — Supplementary Information [file 41467_2020_19385_MOESM1_ESM.pdf]

Brightness modulations of our nearest terrestrial planet Venus reveal atmospheric super-rotation rather than surface features

Y. J. Lee[1], A. García Muñoz[1], T. Imamura[2], M. Yamada[3], T. Satoh[4], A. Yamazaki[4], and S. Watanabe[5]

[1]Technische Universität Berlin, Berlin, Germany
[2]GSFS, Univ. of Tokyo, Kashiwa, Japan
[3]Planetary Exploration Research Center (PERC), Narashino, Japan
[4]Institute of Space and Astronautical Science (ISAS/JAXA), Sagamihara, Japan
[5]Hokkaido Information University, Ebetsu, Japan

October 9, 2020

Supplementary Information

a

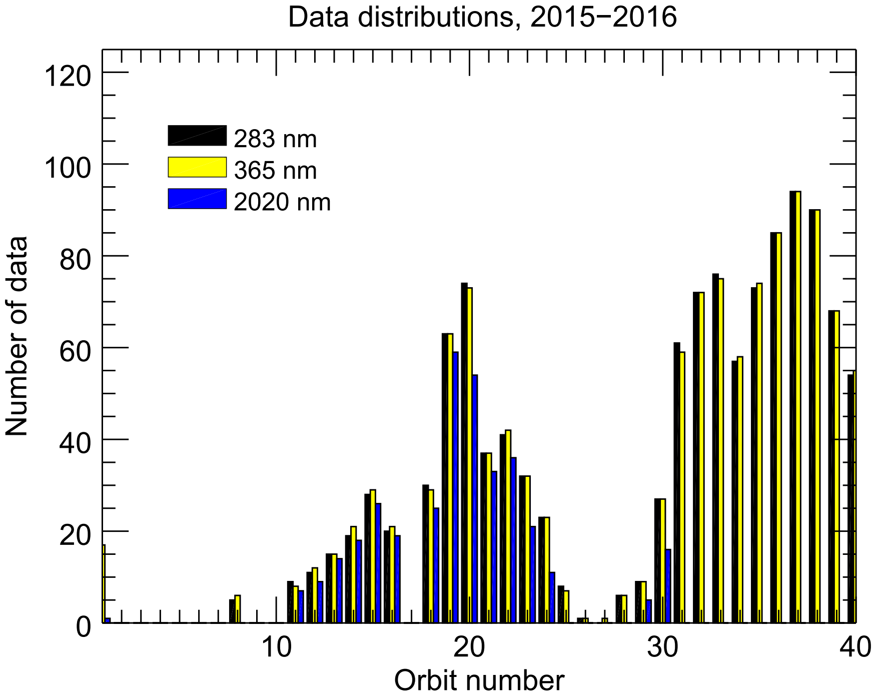

b

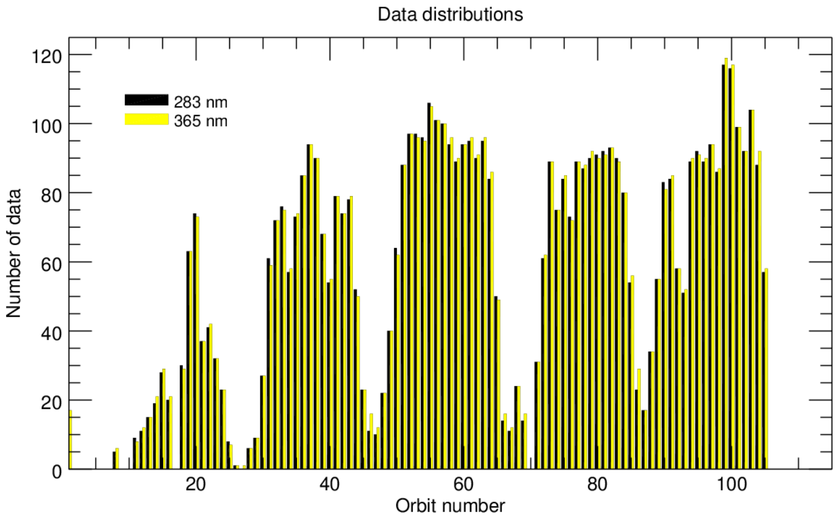

Supplementary Figure 1: Distribution by orbit of the selected full-disk images. **a** UV and NIR images in 2015–2016. **b** UV images until Jan 2019 (orbits 1–105).

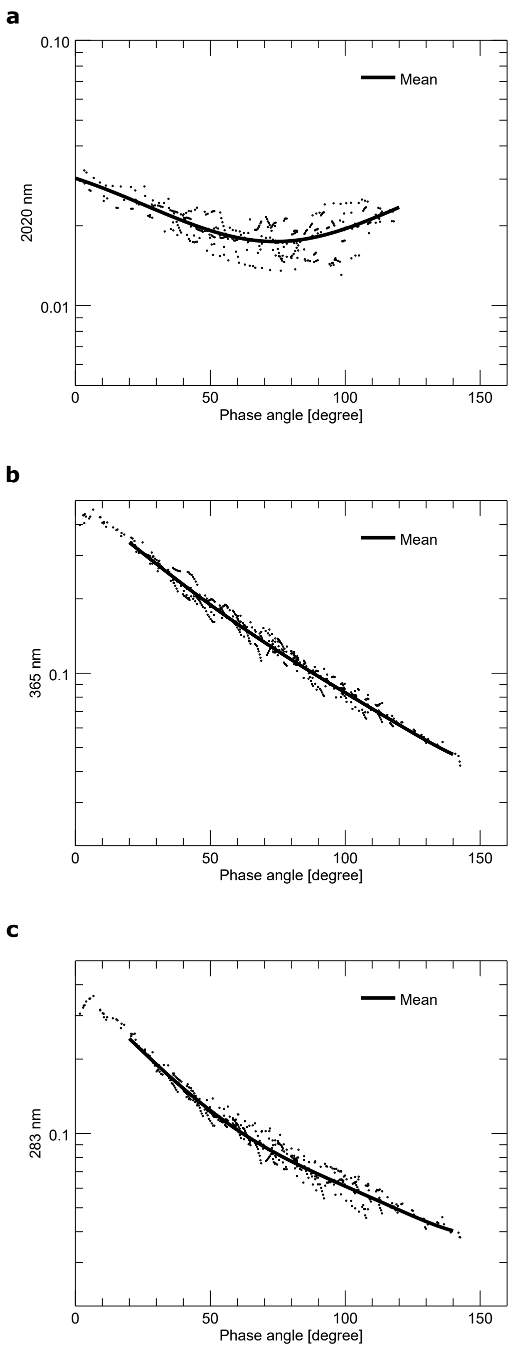

Supplementary Figure 2: Mean phase curves over Mar 31–Oct 29 2016, when both of the cameras UVI and IR2 were operational. For each wavelength, the best-fitting 4th-degree polynomial is shown. **a** 2020 nm in the 0–120° phase angle range. **b** 365 nm and **c** 283 nm in the 20–150° phase angle range. The 0–20° phase angle range is excluded in the UV to avoid the glory feature.

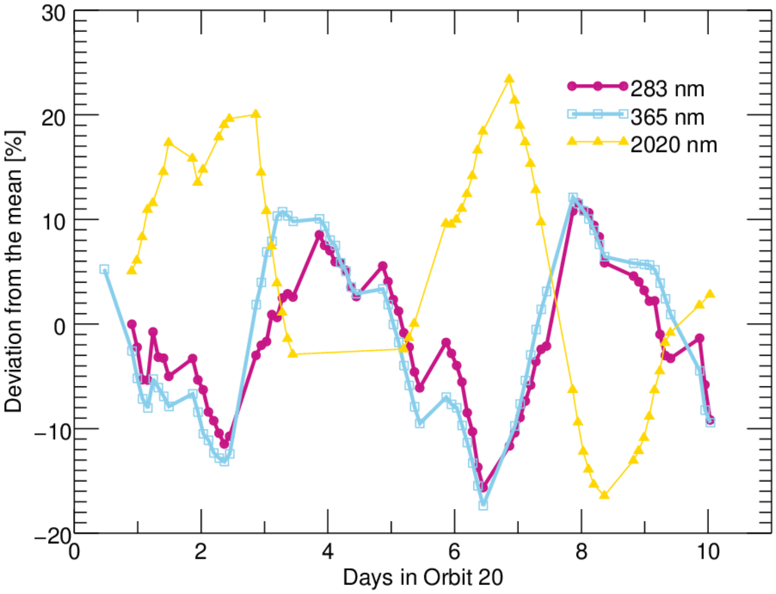

Supplementary Figure 3: Deviations (%) of disk-integrated brightness from the mean phase curve in 2016 during orbit 20 (Figure 1 and Supplementary Figure 2).

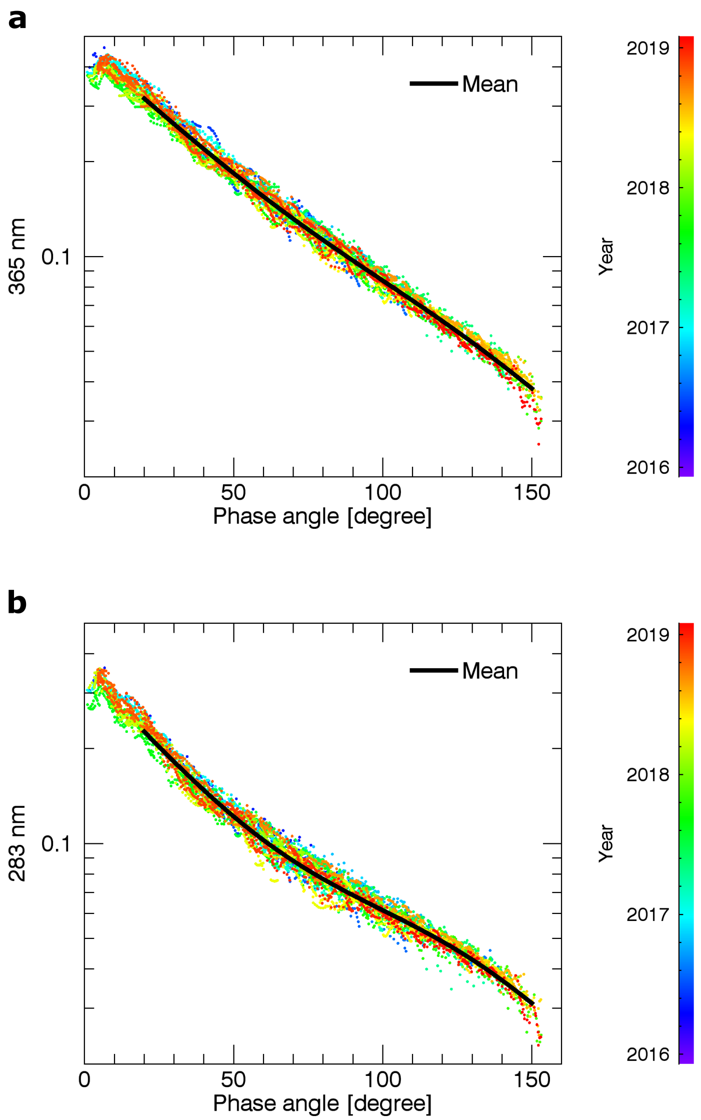

Supplementary Figure 4: Mean phase curves in years 2015–2019 for UV wavelengths. Best-fitting 4th-degree polynomials are shown for **a** 365 nm and **b** 283 nm. Color indicates years. The 0–20° phase angle range is excluded to avoid the glory feature.

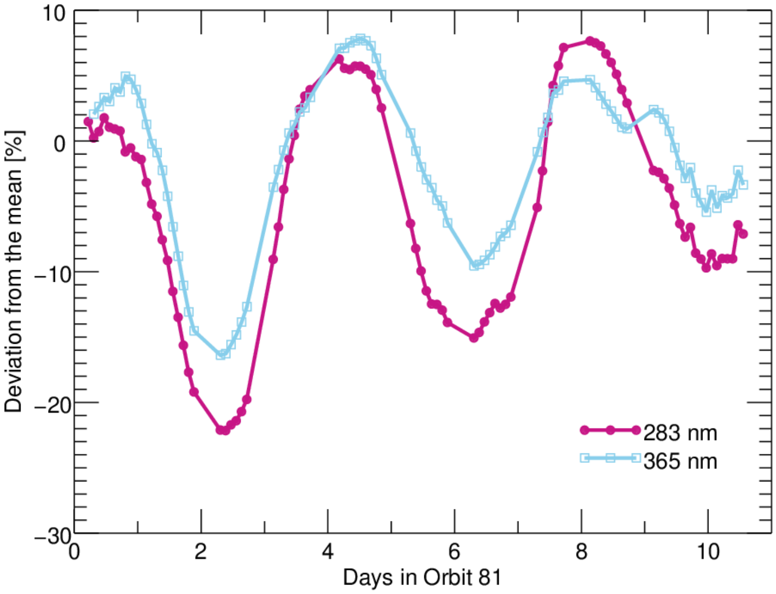

Supplementary Figure 5: Deviations (%) of disk-integrated brightness from the mean phase curves in years 2015–2019 during orbit 81 (Figure 1 and Supplementary Figure 4).

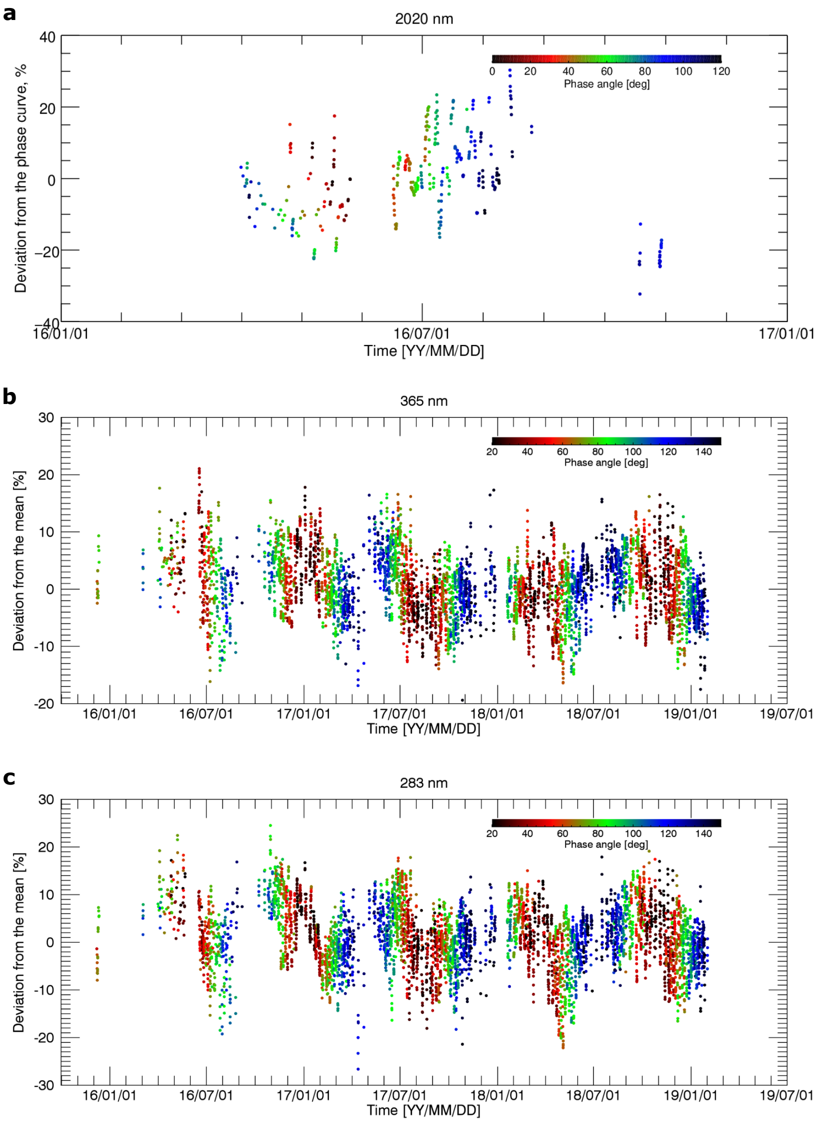

Supplementary Figure 6: Time series of albedo deviations from the mean phase curves shown in Supplementary Figures 2 and 4. **a** 2020 nm in 2016, **b** 365 nm in 2015-2019, and **c** 283 nm in 2015–2019. The 0–20° phase angle range is excluded in the UV to avoid the glory feature.

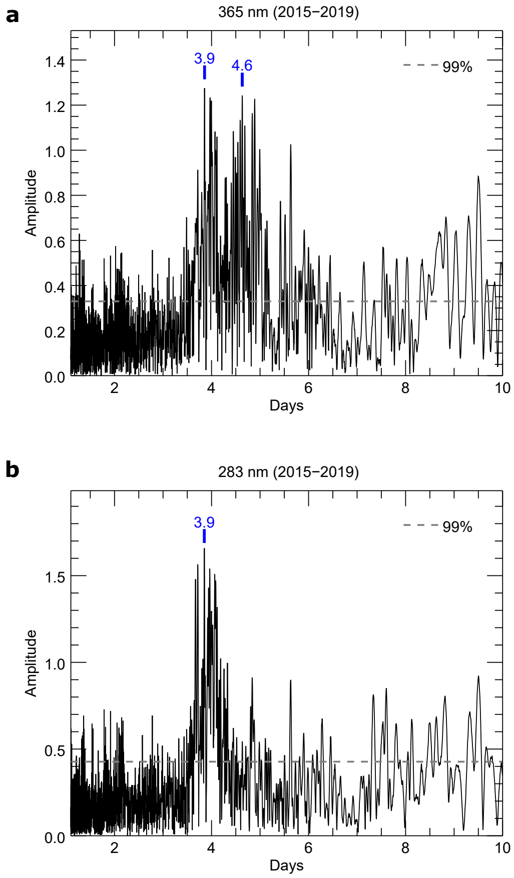

Supplementary Figure 7: Periodograms of UV brighness measurements in years 2015–2019 using the time series of Supplementary Figure 6 for **a** 365 nm and **b** 283 nm. The numbers in blue indicate identified periods in 'Earth Days', and 99% confidence levels are also shown.

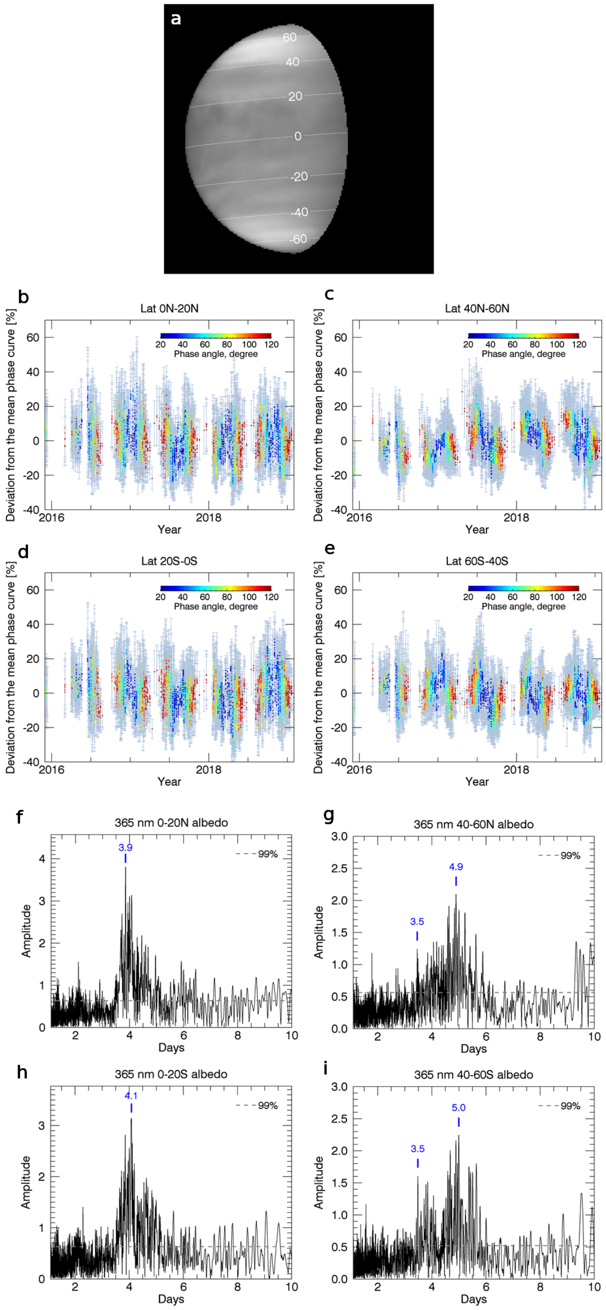

Supplementary Figure 8: Periodicity analysis of disk-resolved photometry at 365 nm. See Supplementary Information Section 1 for details. **a** Example of 365-nm image after photometric correction. Time series of brightness fluctuations at **b** 0–20°N, **c** 40–60°N, **d** 0–20°S, and **e** 40–60°S. Periodograms at **f** 0–20°N, **g** 40–60°N, **h** 0–20°S, and **i** 40–60°S. The numbers in blue indicate the identified periods in ‘Earth Days’, and 99% confidence levels are also shown.

1 Periodicity analysis of disk-resolved photometry

In addition to analyzing the periodicity in the disk-integrated brightness of Venus, we analyzed the periodicity of the brightness over the disk-resolved images. This exercise aims to cross-check the periods obtained from the disk-integrated and disk-resolved treatments, and compare them to previous studies. To that end, the disk-resolved images were photometrically corrected following established procedures[1–4] that remove systematic brightness gradients dependent on incidence (i), emergence (e), and phase angle (α) angles. This is the same method applied to other studies[5,6], whose findings are summarized in Supplementary Table 1. Specifically, we applied the Lambert and Lommel-Seeliger photometric laws, which showed the best performance[2], and used an updated k coefficient at 365 nm,

$$k(\alpha) = 0.270438+0.00269467\times\alpha-(1.19147\times10^{-5})\times\alpha^2-(1.10714\times10^{-7})\times\alpha^3, \quad (1)$$

where α is phase angle in degrees. This k coefficient is derived from the same data set used in our study. An example of photometrically corrected image is shown in Supplementary Fig. 8a. Then, we calculated an average of the corrected brightness in the 0–20°N/S and 40–60°N/S bins for each of the northern and southern hemispheres to take into account hemispheric asymmetry[3,7,8]. We estimated the mean phase curves of brightness at each latitudinal bin, and derived deviations from these mean phase curves as a function of time (Supplementary Figs. 8b–e). Phase angles α >120° were excluded to avoid working with a too small number of valid pixels. Finally, we calculated from the resulting time series the corresponding periodogram for each latitudinal bin (Supplementary Figs. 8f–i). We find a clear impact of latitude on the identified periods: ~4.0-day peaks at low latitudes (0–20°N and S), and ~5-day peaks at mid latitudes (40–60°N and S).

Supplementary Table 1 summarizes the periods found in our analysis for both disk-integrated and disk-resolved photometry, and the periods reported in previous studies (and that typically considered low and middle latitudes separately). Interestingly, the periods from our disk-resolved photometry match better the periods reported in previous studies than those from our disk-integrated photometry do. This result gives us confidence in our general methodology, and suggests that the slight difference of the P_1~3.7 and P_2~4.6 periods discussed in the main text (in particular the latter) with the values reported in other investigations is a consequence of simultaneous spatial and periodic mixing when working with disk-integrated photometry.

Supplementary Table 1: Periodicity of brightness in disk-integrated and -resolved photometry in this study and previous studies.

References	Time	Periodicity (d, Earth days)		
		Disk-integrated (wavelength)	Disk-resolved (365 nm)	
			Low latitudes	Mid latitudes
This study, Fig. 3	2016	3.7 d (283 nm)<br>3.7 and 4.5–4.6 d (365 nm)<br>3.7 and 4.5–4.6 d (2020 nm)		
This study, Supplementary Fig. 7	2016–2018	3.9 d (283 nm)<br>3.9 and 4.6 d (365 nm)		
This study, Supplementary Fig. 8	2016–2018		3.9 d at 0–20°N<br>4.1 d at 0–20°S	4.9 d at 40–60°N<br>5.0 d at 40–60°N
Imai et al. 2019[5], Table 2	90 days, 2017 Jun 18–Sep 16		3.5–5.3 d at 1.75–3.75°S	4.8–5.1 d at 44.25–47.25°S
Nara et al. 2020[6], Section 2.2.3	2018 June		3.6 d at equator	4.0 d at mid latitudes
Del Genio et al. 1982[9], Summary 1, p.413	66 days in 1979		3.94±0.1 d at equator	5.20±0.2 d at 45°
Del Genio et al. 1990[10], Table 2	1979–1985		3.94–5.03 d at equator	5.02-6.00 d at mid latitudes

References

[1] Y. J. Lee, T. Imamura, S. E. Schröder, and E. Marcq. Long-term variations of the UV contrast on Venus observed by the Venus Monitoring Camera on board Venus Express. Icarus, 253:1–15, June 2015.

[2] Y. J. Lee, A. Yamazaki, T. Imamura, M. Yamada, S. Watanabe, T. M. Sato, K. Ogohara, G. L. Hashimoto, and S. Murakami. Scattering Properties of the Venusian Clouds Observed by the UV Imager on board Akatsuki. Astron. J., 154(2):44, August 2017.

[3] Y. J. Lee, K.-L. Jessup, S. Perez-hoyos, D. Titov, S. Lebonnois, J. Peralta, T. Horinouchi, T. Imamura, S. Limaye, E. Marcq, M. Takagi, A. Yamazaki, M. Yamada, S. Watanabe, S. Murakami, K. Ogohara, W. McClintock, G. Holsclaw, and A. Roman. Long-term variations of Venus' 365-nm albedo observed by Venus Express, Akatsuki, MESSENGER, and Hubble Space Telescope. The Astronomical Journal, 158(3):126, aug 2019.

[4] Y. J. Lee, P. Kopparla, J. Peralta, S. E. Schröder, T. Imamura, T. Kouyama, and S. Watanabe. Spatial and Temporal Variability of the 365-nm Albedo of Venus Observed by the Camera on Board Venus Express. Journal of Geophysical Research (Planets), 125(6):e06271, June 2020.

[5] M. Imai, T. Kouyama, Y. Takahashi, A. Yamazaki, S. Watanabe, M. Yamada, T. Imamura, T. Satoh, M. Nakamura, S.-y. Murakami, K. Ogohara, and T. Horinouchi. Planetary-scale variations in winds and UV brightness at the Venusian cloud top: Periodicity and temporal evolution. J. Geophys. Res. (Planets), 124, Aug 2019.

[6] Y. Nara, T. Imamura, K. Masunaga, Y. J. Lee, N. Terada, K. Yoshioka, A. Yamazaki, K. Seki, I. Yoshikawa, M. Yamada, and S. Watanabe. Vertical Cou-

pling Between the Cloud-Level Atmosphere and the Thermosphere of Venus Inferred From the Simultaneous Observations by Hisaki and Akatsuki. Journal of Geophysical Research (Planets), 125(3):e06192, March 2020.

[7] T. Horinouchi, T. Kouyama, Y. J. Lee, S.-y. Murakami, K. Ogohara, M. Takagi, T. Imamura, K. Nakajima, J. Peralta, A. Yamazaki, M. Yamada, and S. Watanabe. Mean winds at the cloud top of Venus obtained from two-wavelength UV imaging by Akatsuki. Earth, Planets, and Space, 70:10, January 2018.

[8] P. Kopparla, Y. J. Lee, T. Imamura, and A. Yamazaki. Principal components of short-term variability in the ultraviolet albedo of venus. Astron. Astrophys., 626:A30, 2019.

[9] A. D. Del Genio and W. B. Rossow. Temporal variability of ultraviolet cloud features in the Venus stratosphere. Icarus, 51:391–415, August 1982.

[10] A. D. Del Genio and W. B. Rossow. Planetary-scale waves and the cyclic nature of cloud top dynamics on Venus. J. Atmospheric Sci., 47:293–318, February 1990.
