# Supplementary material for: Brightness modulations of our nearest terrestrial planet Venus reveal atmospheric super-rotation rather than surface features
Source: Nat Commun. 2020 Nov 12;11:5720. doi: 10.1038/s41467-020-19385-6 (PMC7665209; doi:10.1038/s41467-020-19385-6)
Supplement: Supplementary file 3 — Description of Additional Supplementary Files [file 41467_2020_19385_MOESM3_ESM.docx]

**Description of Additional Supplementary Files**

File Name: Supplementary Data 1
Description: Disk-integrated brightness of Venus at 283 nm in 2015-2019

File Name: Supplementary Data 2
Description: Disk-integrated brightness of Venus at 365 nm in 2015-2019

Supplementary Dataset 3
Description: Disk-integrated brightness of Venus at 2020 nm in 2016

File Name: Supplementary Movie 1
Description: Temporal variability in the phase-resolved albedo at 283, 365, and 2020 nm in 2016

File Name: Supplementary Movie 2
Description: Temporal variability in the phase-resolved albedo at 283 and 365 nm in 2015-2019
